# Patterns of *Fasciola hepatica* infection in Danish dairy cattle: implications for on-farm control of the parasite based on different diagnostic methods

**DOI:** 10.1186/s13071-018-3248-z

**Published:** 2018-12-29

**Authors:** Nao Takeuchi-Storm, Matthew Denwood, Heidi Huus Petersen, Heidi Larsen Enemark, Anna-Sofie Stensgaard, Mita Eva Sengupta, Nicola Jane Beesley, Jane Hodgkinson, Diana Williams, Stig Milan Thamsborg

**Affiliations:** 1 Research Group for Veterinary Parasitology, Department of Veterinary and Animal Sciences, University of Copenhagen, Dyrlægevej 100, DK-1871 Frederiksberg C, Denmark; 2 Section for Animal Welfare and Disease Control, Department of Veterinary and Animal Sciences, University of Copenhagen, Grønnegårdsvej 8, DK-1870 Frederiksberg C, Denmark; 3 National Veterinary Institute, Technical University of Denmark, Kemitorvet, DK-2800 Kongens Lyngby, Denmark; 4 Research Group for Animal Health, Department of Animal Health and Food Safety, Norwegian Veterinary Institute, P.O. Box 750, Sentrum, NO-0106 Oslo, Norway; 5 Center for Macroecology, Evolution and Climate, The Natural History Museum of Denmark, University of Copenhagen, Universitetsparken 15, DK-2100 København Ø, Denmark; 6 Department of Infection Biology, Institute of Infection and Global Health, University of Liverpool, Liverpool Science Park IC2, 146 Brownlow Hill, Liverpool, L3 5RF UK

**Keywords:** *Fasciola hepatica*, Transmission, Dairy, Diagnostic, ELISA, Denmark

## Abstract

**Background:**

Bovine fasciolosis is an economically important livestock disease in Europe, and represents a particular challenge for organic farms, where cattle are grazed extensively and the use of anthelmintic is limited. A two-year longitudinal study was conducted on two conventional and two organic Danish dairy farms to examine the current temporal trend of *F. hepatica* infection on-farm, and to gather data of practical relevance for parasite control. Data were collected both at the herd and individual level using currently available diagnostic methods: a commercial serum antibody ELISA, a commercial copro-antigen ELISA, faecal egg counts, and monthly bulk tank milk (BTM) ELISA. The temporal patterns (animal age, farm-level temporal trends and seasonality) in the animal-level test results were analysed by generalised additive mixed models (GAMM).

**Results:**

Patterns of infection differed substantially between the farms, due to different grazing management and anthelmintic use. However, animals were first infected at the age of 1.5–2 years (heifers), and most at-risk animals sero-converted in autumn, suggesting that summer infections in snails prevail in Denmark. Our results also suggest that the lifespan of the parasite could be over 2 years, as several cows showed signs of low grade infection even after several years of continuous indoor housing without access to freshly-cut grass. The serum antibody ELISA was able to detect infection first, whereas both copro-antigen ELISA and faecal egg counts tended to increase in the same animals at a later point. Decreasing BTM antibody levels were seen on the two farms that started anthelmintic treatment during the study.

**Conclusions:**

While important differences between farms and over time were seen due to varying grazing management, anthelmintic treatment and climatic conditions, the young stock was consistently seen as the main high-risk group and at least one farm also had suspected transmission (re-infection) within the lactating herd. Careful interpretation of test results is necessary for older cows as they can show persistent infections several years after exposure has stopped. Rigorous treatment regimens can reduce BTM ELISA values, but further research is needed to develop a non-medicinal approach for sustainable management of bovine fasciolosis.

**Electronic supplementary material:**

The online version of this article (10.1186/s13071-018-3248-z) contains supplementary material, which is available to authorized users.

## Background

The trematode *Fasciola hepatica* raises substantial concerns for the cattle industry due to reduced productivity, increased susceptibility to other diseases and interaction with diagnostic tests for bovine tuberculosis [1–4]. Despite efforts to develop a vaccine against the parasite, control of bovine fasciolosis still relies largely on preventive measures such as drainage, avoiding or fencing off snail habitats, and anthelmintic treatment [3, 5]. Increasing anthelmintic resistance [2, 6] further emphasise the importance of responsible and efficient use of anthelmintics, i.e. in combination with grazing management.

To successfully control fasciolosis on a dairy farm, it is crucial to identify which pasture is the source of infection. This is most often achieved by taking samples from representative groups of animals in different age groups grazing identified pastures, and analysing them either by faecal egg counts or by ELISA to detect antibodies in serum or milk, depending on which age group is tested [5]. However, careful interpretation of the results is needed, because *F. hepatica* infection is known to be seasonal, has a long prepatent period and each diagnostic test provides different information about the infection. Copro-antigen ELISA is a relatively new diagnostic technique that can detect infections at least five weeks after uptake [7, 8]. Yet, sensitivity and specificity vary depending on field conditions [9–12], and interpretation of copro-antigen ELISA results from the field is still unclear. Additionally, pasture can be examined for potential snail habitats (wet areas) to identify the source of infection [5], because transmission is unlikely to occur if snail habitats are absent on the pasture in question. In fact, presence of the intermediate host snails, G*alba truncatula*, has been described as the most significant factor in predicting the herd-level exposure for *F. hepatica* [13]. Identification of snail habitats and intermediate host snails, is thus an important part of on-farm fasciolosis control, although the procedure can be time-consuming and requires specialized taxonomical skills or molecular tools to correctly identify the *G. truncatula* snails [3].

Recent studies have suggested an altered transmission pattern of *F. hepatica*, both spatially and temporally, as a consequence of changing climatic conditions [14]. Extended geographical distribution and increased prevalence have already been observed in recent years in some parts of Europe, attributed to altered temperature and rainfall patterns [15–18]. Concerns over future impacts of climate change on the seasonality have also been raised; increased outbreaks due to winter infection and decreased summer infection are to be expected in bi-seasonal transmission areas [19]. Despite the increasing concerns, only a few studies have investigated the temporal patterns of *F. hepatica* infection in animals on individual farms in recent years [20, 21]. Transmission patterns were extensively studied in 1970s in Denmark, showing that winter infection occurred in some years, but the major part of the total fluke population could be ascribed to summer infection of the snails [22, 23]. Since then, no studies have been conducted in Denmark to assess if the transmission patterns have changed. In addition, change in transmission patterns may be attributed to a recent shift in production systems, i.e. an increase in organic production. In 2017, the number of organic cattle in Denmark was approx. 200,000, corresponding to ten times more than in 1995 [24]. Compared to Sweden, where all cattle have to graze regardless of whether they are organic or conventional [16], only organic farms are obliged to graze all stock in Denmark, and conventional farms with zero-grazing are not uncommon [25]. The parasitic challenge is greater in farms with outdoor access [26, 27] and the prevalence of *F. hepatica* is higher in organic than conventional farms in Denmark [17]. Additionally, the withdrawal period for veterinary medicines including anthelmintics are twice as long for organic farms [28] and minimum use of veterinary medicines is an important concept for organic producers [29]. Integrated control, e.g. by grazing management is therefore desirable [26], and updated knowledge about on-farm *F. hepatica* transmission is crucial for development of such control strategy. Moreover, a pragmatic approach is required for implementation of on-farm control strategies. Questions such as “can cattle get re-infected?”, “how long do liver flukes live in cattle?” and “how long do the antibodies last after treatment?” are often asked by the cattle producers and veterinary practitioners, but are insufficiently addressed in the current literature.

The aim of this longitudinal observational study was to explore the temporal patterns of infection on four Danish dairy farms (conventional and organic) in terms of age groups, individual and herd-level diagnostic methods, and seasonality, including relative importance of summer and winter infection of snails. Each farm was examined extensively including grazing and treatment strategies to elucidate the similarities and differences of the transmission of *F. hepatica* due to varying farm-specific management. Ultimately, we aimed to generate data that can be translated into suggestions for improved practical and realistic guidelines for diagnosis and control of fasciolosis.

## Methods

### Farm selection and background

**Fig. 1:**
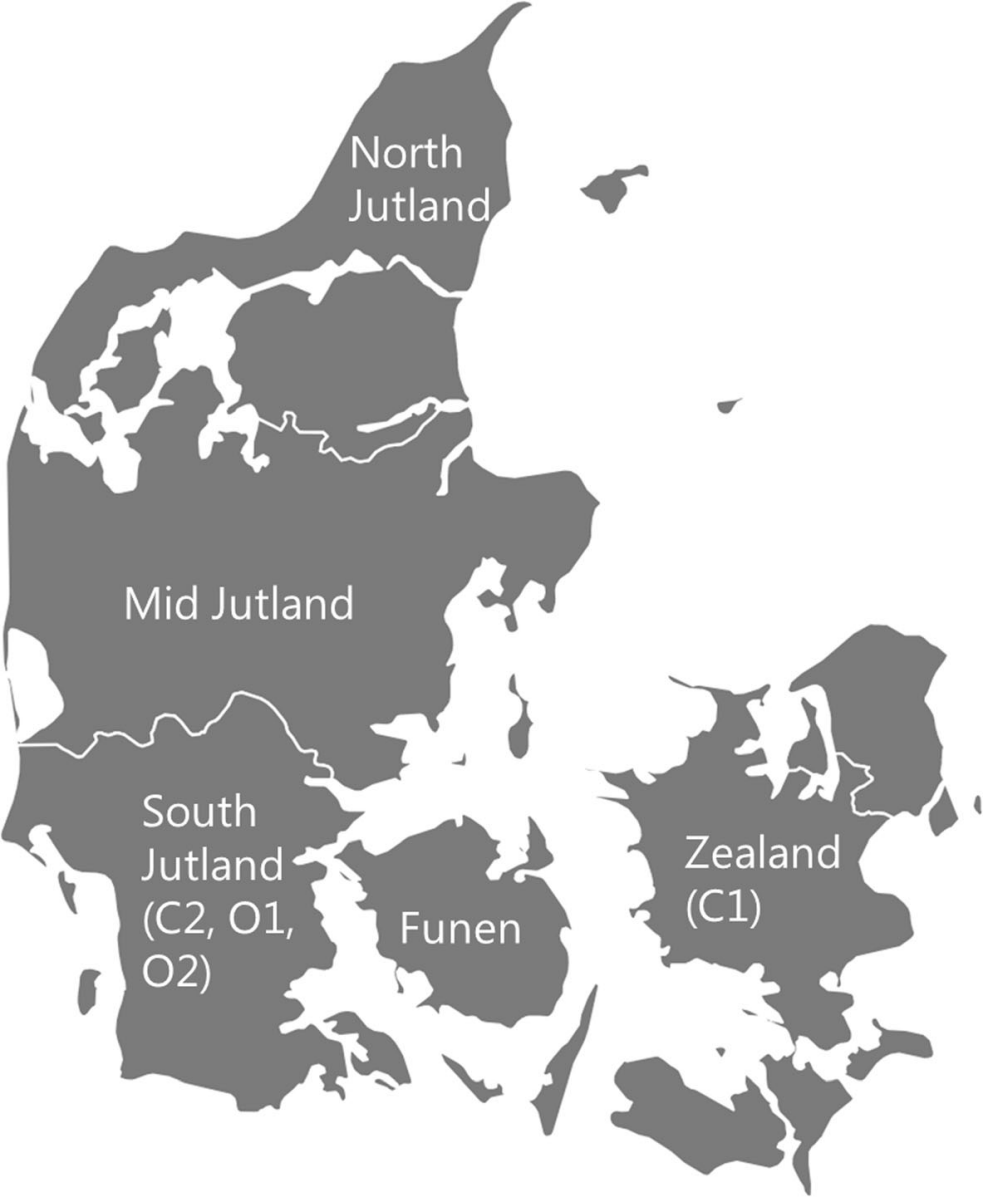
Map of Denmark, showing the regions and locations of the four farms that participated in the study

**Fig. 2:**
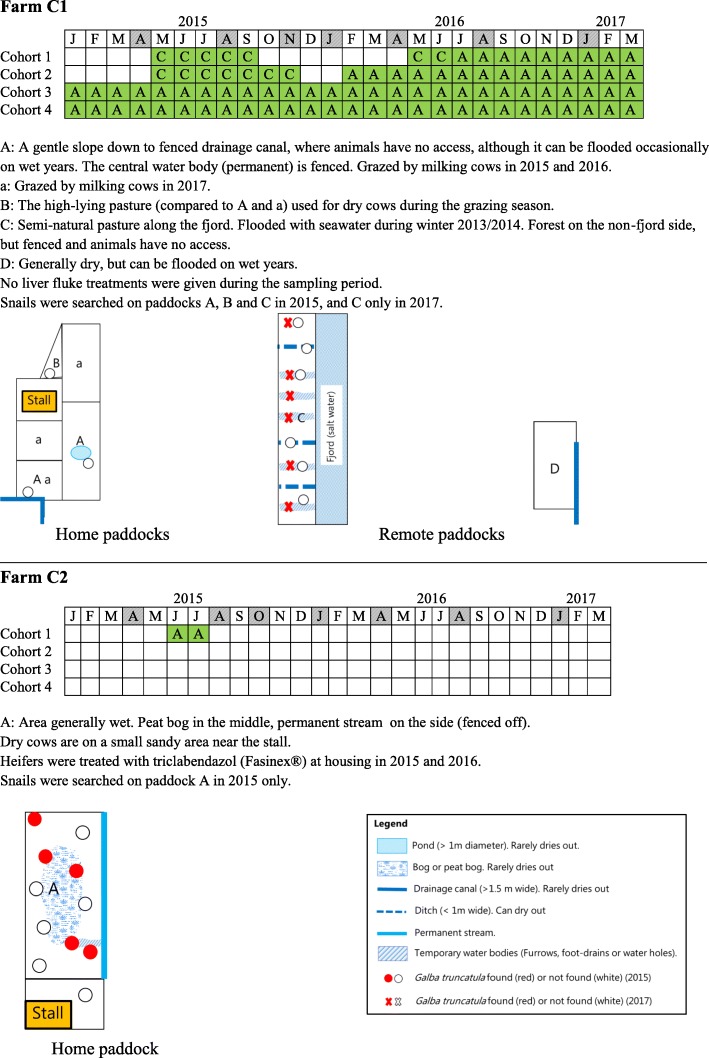
Schematic map and Gantt chart of grazing periods (grey shaded, time of sampling; green shaded, grazing; pasture areas are indicated by capital letters), pasture characteristics (refer to the common map legend) and treatment against *Fasciola hepatica* on farms C1 and C2, 2015–2017

**Fig. 3:**
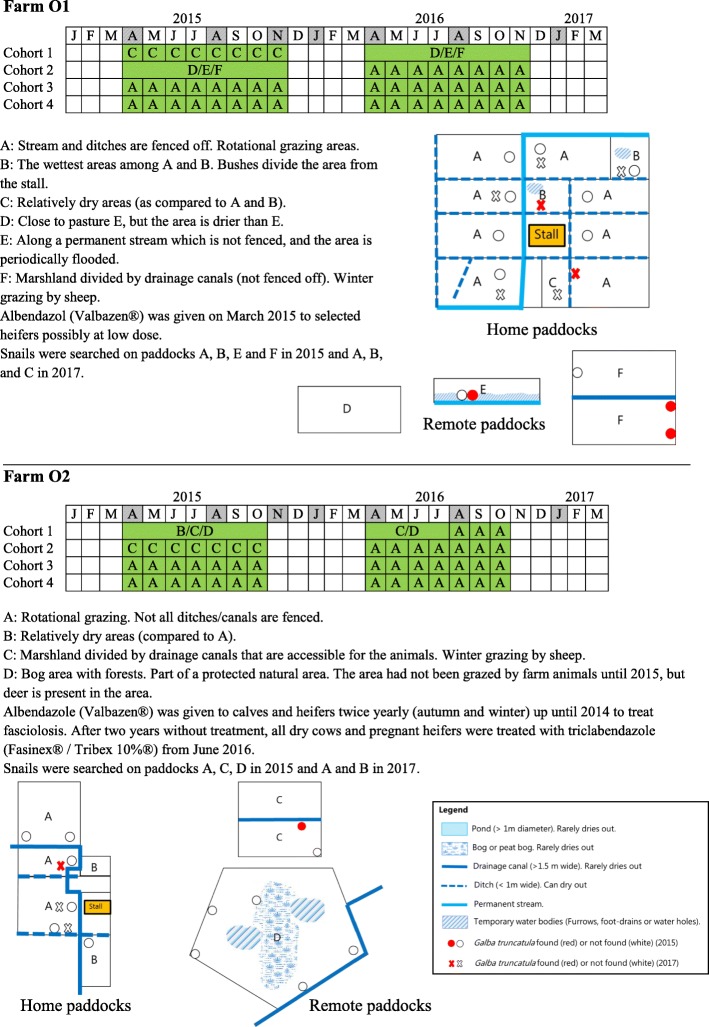
Schematic map and Gantt chart of grazing periods (grey shaded, time of sampling; green shaded, grazing; pasture areas are indicated by capital letters), pasture characteristics (refer to the common map legend) and treatment against *Fasciola hepatica* on farms O1 and O2, 2015–2017

Potentially suitable study farms were identified from our previous study [25] in conjunction with SEGES (part of the Danish Agricultural Advisory Service run by the Danish Agriculture and Food Council) and Økologisk landsforening (National Organic Association) based on likely farmer compliance and interest in participating in the study for the entire planned study period of 2015–2017. From these, two conventional (C1 and C2) and two organic (O1 and O2) farms were selected based on known infection status as judged by bulk tank milk (BTM) ELISA values and high levels of liver condemnation at slaughter during the period 2011–2014 (Table 1). Farm C1 was located on the Island of Zealand, while Farm C2, O1 and O2 were located within 30 km of one another in South Jutland (Fig. 1). Danish organic rules abide by Council Regulation (EC) No. 834/2007 of 28 June 2007 on organic production and labelling of organic products and repealing Regulation (EEC) No. 2092/91. All selected farms had all-year calving, and automatic milking systems were used on farms O2 and C2. Further farm-specific details are given below together with a simple schematic plot and Gantt chart for each farm (Figs. 2 and 3). The participating farmers were regularly updated with our findings and consultation meetings were held twice (halfway and end of the study period) together with their consultants and veterinary practitioners.

**Table 1:** Summary of data used as inclusion criteria for the 4 farms in the study

Farm	Year	No. of heifers	No. of cows	Total no. of cattle	Liver condemnation (%)	BTM ELISA value (S/P%)^a^
C1	2011	72.5	176.5	314	6.2	–
	2012	65.5	184.5	303	21.3	–
	2013	65	187.5	315	30.0	–
	2014	63	183.5	312	18.6	179.3
C2	2011	103	135	292	8.3	–
	2012	98.5	145	300	11.9	–
	2013	105.5	144	314	16.1	–
	2014	111	149	331	19.4	181.2
O1	2011	145	172	367	2.6	–
	2012	141	168	362	7.6	–
	2013	124	174.5	354	33.3	–
	2014	172	183	425	23.3	221.4
O2	2011	90.5	113.5	251	18.1	–
	2012	97.5	124	275	32.6	–
	2013	111	131.5	282	27.7	–
	2014	113	133.5	285	38.1	206.9

^a^by IDEXX ELISA test (cut-off is 30 and ≥ 150S/P% is considered high)

#### Farm C1

Calves are turned out when they are 5–9 months-old on a pasture away from the stall (Fig. 2 C1-D). Animals of 9 to 12 months of age are all grazed with larger heifers on pasture, which is located along a fjord with seawater (Fig. 2 C1-C). This pasture is shared with two beef farms with no previous anthelmintic treatment for liver fluke. The heifers are divided into five different groups based on age, and every five weeks during the grazing season, one group at a time is housed for insemination and given ectoparasitic treatments (Noromectin® pour-on, Biovet ApS, Denmark and Butox® 7.5% Pour-on, MSD Animal Health A/S, Denmark). This means that some heifers graze only for five weeks, while some others may graze for the whole grazing season. The longest grazing period for the heifers during the study period was mid-May to mid-November. Dry cows graze from 6 weeks before calving on a dry, high-lying (high elevation) pasture near the farm house (Fig. 2 C1-B). The area is available for grazing from May to November. Milking cows are housed in a deep litter stall with access to pasture 24 hours a day and all year around. The pastures for milking cows are found on terrain that has a slight slope towards a drainage canal that cannot be accessed by the animals (Fig. 2 C1-A). The milking cows are furthermore prevented from having access to a fenced-off waterhole in this paddock. No treatments for liver fluke were given during the study period, but triclabendazole (Tribex 10%® ScanVet Animal Health A/S, Denmark) was given at housing to all young animals that grazed on the fjord pasture (Fig. 2 C1-C) in 2017. Animals are mostly Danish Holsteins (DH) with some cross-breeds.

#### Farm C2

Heifers are the only grazing animals on this farm (i.e. all milking cows are housed year round). The pasture for heifers is on wet, low ground with a central peat bog (Fig. 2 C2-A). Dry cows utilise a sandy exercise yard near the stall, which is not considered to be suitable snail habitat because it is consistently dry. Before commencement of our study, the farmer treated only some of the heifers with triclabendazole (Fasinex240®, Elanco, Denmark) every November. However, this management changed part-way through the study (in 2015) so that all heifers in the first and second trimester were routinely treated with triclabendazole following housing. The grazing period for heifers is typically early June to mid-October, although 90% of animals were housed in late July 2015 due to low feed availability. Most animals are DH and the rest are cross-breeds.

#### Farm O1

The calves are turned out when they are 4 months-old on a dry permanent pasture with access to a stall close to the farm house (Fig. 3 O1-C). They have access to feed *ad libitum*. Young heifers are grazed in two separate areas away from the farm house (Fig. 3 O1-D and E). Heifers to be inseminated graze together with dry cows close to the farm house on a wet pasture (Fig. 3 O1-B). They are fed once a day in the stall. Once pregnant, heifers are moved to a pasture on reclaimed marshland (freshwater meadows) (Fig. 3 O1-F). During winter, this pasture is grazed by sheep who are treated twice yearly with triclabendazole. Milking cows graze around the farm house rotationally, and some of these pastures can be very wet depending on weather and season (Fig. 3. O1-A). Albendazole (Valbazen®, Orion Pharma Animal Health A/S, Denmark; unknown dosage) was applied to a few selected heifers due to sub-optimal weight gain during 2015. Six treated animals were included at the first sampling, but high copro-antigen levels and faecal egg counts were observed in these animals. The treatment dosage was therefore assumed to be targeted against nematodes rather than liver fluke. The grazing period during our study was early April to late November. Most animals on the farm are cross-breeds mainly with DH, and the rest are Danish red and DH.

#### Farm O2

Calves are turned out on pasture near the farm house at 4 months-old (Fig. 3 O2-B). Older calves and heifers are grazed in two separate areas (Fig. 3 O2-C and D). One area is a reclaimed marshland (freshwater meadow), where sheep graze during winter (Fig. 3 O2-C) and are treated as above (O1). The other area is a bog, which is part of a protected natural area with forests and in which red deer (*Cervus elephus*) and roe deer (*Capreolus capreolus*) are regularly observed (Fig. 3 O2-D). This bog area was granted to the farmer for grazing from 2015; the area had not been grazed by farm animals previously. Milking cows graze around the farm house in pastures which are rotated between years (Fig. 3 O2-A). Some of these pastures are low-lying and consistently wet. Albendazole was given to calves and heifers twice yearly (autumn and winter) up until 2014 to treat fasciolosis. After two years of no treatment and following the results of the first four sampling events, the farmer started treating all dry cows and pregnant heifers with triclabendazole from June 2016. The grazing period during the study was mid-April to mid-October. Most animals on the farm are DH and the rest are cross-breeds.

### On-farm animal sampling and other data sources

Each farm was visited seven times at the following time points: turn-out (spring) 2015, summer 2015, housing (autumn) 2015, winter 2015/2016, turn-out (spring) 2016, summer 2016, and winter 2016/2017 (Figs. 2 and 3). At the first sampling event, animals were enrolled into the study within four age groups as follows: calves with a first grazing season in 2015, heifers that had first grazed in 2014, primiparous cows, and multiparous cows. These animals were selected randomly at farm O1, but on the other farms animals were selected for convenience by the farmers. At each time point, blood and faecal samples were collected from each cohort of animals. If an enrolled animal was slaughtered during the study, it was replaced by another animal within the same age group at the next sampling time point. In total, 229 individual animals were sampled, equating to 1078 faecal samples that were analysed by serum ELISA, 1170 by copro-antigen ELISA, and 1172 by sedimentation, respectively (Table 2). Of these, 39 animals (12 and 27 animals from farms C2 and O2, respectively) were treated with triclabendazole during the study period. Blood samples from primiparous and multiparous cows were not taken on the first visit due to logistical difficulties. The summer samples from calves and heifers from O2 were not taken due to lack of safe handling facilities on pasture.

**Table 2:** Summary of the number of animals sampled at each time point

		Spring 2015	Summer 2015	Autumn 2015	Winter 2015/16	Spring 2016	Summer 2016	Winter 2016/17
C1	Cohort 1	11	11	11	11	11	11	8
	Cohort 2	11	11	11	11	11	11	10
	Cohort 3	11^a^	11	11	11	11	11	9
	Cohort 4	11^a^	11	11	11	11	11	9
C2	Cohort 1	11	11	10	10	13	13	13
	Cohort 2	11	11	11	11	12	10	8
	Cohort 3	11^a^	10	10	10	9	9	6
	Cohort 4	13^a^	12	13	12	8	7	6
O1	Cohort 1	11	11	11	11	12	12	11
	Cohort 2	11	11	11	12	11	10	9
	Cohort 3	11^a^	11	10	10	10	10	8
	Cohort 4	11^a^	11	11	11	11	10	8
O2	Cohort 1	11	2^b^	11	11	11	8	8
	Cohort 2	11	8^b^	12	12	12	14	13
	Cohort 3	11^a^	9	10	10	10	11	8
	Cohort 4	11^a^	13	12	12	12	8	8

^a^Blood samples from primiparous and multiparous cows were not taken on the first visit due to logistic reasons

^b^The summer samples from calves and heifers from O2 were not taken due to lack of safe handling facilities on pasture

Blood samples were centrifuged at 1450 g for 10 min within 24 h of collection and serum was stored at -20 °C until analysis. Additionally, BTM collected as part of the mandatory milk control scheme laid by the Ministry of Food, Agriculture and Fisheries in accordance with EU regulation on hygiene of food stuff (EC No. 853/2004) was stored frozen once a month at a commercial laboratory and periodically (every 6 to 12 months) forwarded by courier to our laboratory. BTM were centrifuged at 1000g for 20 min to separate the fat and the whey was kept at -20 °C until analysis.

In addition to the on-farm data, register data regarding birth date, calving dates, lactation number, liver condemnation at slaughter of each animal present on the study herds during 2014–2016 was extracted from the Danish Cattle Database (DCD). Furthermore, treatment history over the period 2014–2016 for the relevant farms was extracted from the Danish centralised register for sales of veterinary medicines (VetStat, The Danish Veterinary and Food Administration (DVFA), Ministry of Environment and Food) and from the farmer’s own paper-based records where necessary. Monthly climate data for the period 2015–2017 as well as the 30-year average air temperature and precipitation records over the period 1960–1990 were obtained from the online archive of the Danish Meteorological Institute [30].

### Diagnostic tests

#### Faecal egg count (FEC) by sedimentation

Five-gram faecal samples were examined by sedimentation technique for presence of trematode eggs [31]. According to Rapsch et al. [32], this technique has a sensitivity and a specificity of 69% and 98%, respectively, when 10 g faecal samples are analysed.

#### Serum and bulk tank milk ELISA

Anti-*F. hepatica* antibody levels were assessed in individual serum samples and monthly BTM by a commercial ELISA kit (IDEXX *Fasciola* verification test®, IDEXX Laboratories, Hoofddorp, the Netherlands) in duplicate according to the manufacturer’s instructions. The results were expressed as sample to positive ratio (S/P%), and it was considered positive if the average of the duplicates was S/P% > 30 (following the manufacturer’s recommendations). The reported sensitivity and specificity of this commercial test using bovine sera are 88–98%, and 84–98%, respectively [12, 32, 33].

#### Copro-antigen ELISA

Two grams of faecal samples were frozen at -20 °C until analysis by a commercial ELISA kit (Bio K210, Bio-X Diagnostics, Rochefort, Belgium). The procedure followed manufacturer’s instructions with the following modifications. The dilution buffer was added to defrosted faecal samples and kept at 5 °C overnight [10]. Incubation with tetramethylbenzidine (TMB) chromagen was extended from 10 to 30 min in order to improve the discrimination between positive and negative samples. Each sample was tested in duplicates and faecal samples from five, 1–3 month-old in-door reared calves from a conventional Danish dairy farm were pooled and included as negative faeces control in each plate. The ELISA results were expressed as ELISA unit (EU). The sample was considered positive if the average EU of duplicates was equal to or above the custom cut-off value (1.89 EU) calculated as the mean EU of all negative faeces controls plus 3-fold standard deviation of the mean. The reported sensitivity and specificity of this test are 77–87% and 99%, respectively [10, 11].

### Analysis of longitudinal data

ELISA results were calculated in Microsoft Excel 2010 (Version 14.0) and all diagnostic test data were then imported into R [34] and merged with data from DCD using the animals’ unique identification numbers. Graphic visualisations of the raw data for each animal along with monthly trends in BTM results were made using the *ggplot2* package [35]. Samples taken after anthelmintic treatment were omitted from the dataset used for drawing graphs. Correlations between the average serum antibody levels of all milking cows and the antibody levels in the BTM taken closest to the sampling date (within 1–24 days) were quantified by Pearson’s correlation coefficient.

The associations between observed individual-animal results and the age of the animals, seasonality, and longer-term temporal trends at the time of sampling were estimated using a generalised additive mixed model (GAMM) implemented using the *mgcv* package [36]. This statistical modelling method allows smoothed spline functions to be fit to linear predictors without imposing any predetermined form on the relationship, and therefore allows the relationship between the diagnostic test result and each of the linear predictors given above to be estimated in a multivariable model that also accounts for the other, highly correlated, predictor variables. Serum ELISA and coproantigen ELISA were log-transformed and used as a linear response variable. A quasi-Poisson distribution was used to model the response variable of FEC (count per 5 g) in order to allow for the over-dispersion that was assumed to be present based on previous experience with FEC data. This quasi-Poisson distribution was used instead of a Poisson model using an observation-level random effect because the latter model failed to converge for two farms, and in place of the more commonly used negative binomial distribution that is not implemented for the GAMM function. Each combination of diagnostic test and farm was modelled independently. Seasonality was incorporated in the model using a standard sine wave method with period set to 365 days and linear transformations of phase and amplitude estimated as linear effects. Longer-term temporal effects were estimated using a smoothing spline based on the sampling date. The effect of animal age was estimated using a smoothing spline based on the age of the animal at the time of sampling. Individual animal ID was included as a random effect in order to control for repeated sampling within animals. Finally, a dichotomous variable reflecting recent treatment record (treated within 180 days from the sampling date or not) was also included as a fixed effect for farms C2 and O2. Model fit was assessed by inspecting residual versus fitted plots and quantile-quantile plots of residuals. In addition, predictions for all animals with more than 3 samples were selected and the residuals for these observations were plotted against the age of the animal to check for any residual temporal autocorrelation. Final model results were visualised by estimating the predicted hypothetical values (and associated 95% confidence intervals) for each of varying animal age, season, and date given fixed values for the other predictors.

#### Changes in test values post-treatment

The treatment response measured by diagnostic test results were summarised graphically for those pre- and post-treatment samples that were available. Number of days since treatment was calculated and changes in test values were then visually assessed for each diagnostic method.

#### Comparison of diagnostic test results

Pairwise agreement among the three diagnostic tests was assessed by Cohen’s kappa using the *irr* package [37, 38]. In addition, agreement between liver condemnation results at slaughter and the results of any diagnostic tests that were taken within 60 days of slaughtering was also assessed using Cohen’s kappa. The interpretation of the Kappa values was as follows: “very good” (> 0.8); “good” (> 0.6 and ≤ 0.8), “moderate” (> 0.4 and ≤ 0.6), “fair” (< 0.2 and ≤ 0.4), and poor (≤ 0.2) [39].

### Snail surveys and detection of *Fasciola hepatica* in snails

Farms were visited in June and October 2015 and again in October 2017 to search for *G. truncatula* snails on pastures where *F. hepatica* transmission was suspected to take place. Due to the large size of the areas that were used for grazing, snail sampling was done in a qualitative manner, by screening all surface water bodies present at the time of sampling. Permanent water bodies were searched by scooping and by visual inspection of the moist/muddy zones at water body edges. The more transient surface water bodies (i.e. moist areas and furrows) were inspected visually and snails were picked with tweezers. All retrieved snails were kept alive in plastic containers with water, and transported back to the laboratory, where they were identified to species level based on morphological characteristics [40, 41]. The *G. truncatula* snails collected in 2015 were furthermore subjected to light-induced shedding for cercarial parasite stages, and finally crushed and dissected to search for patent and pre-patent stages of *F. hepatica* in the snail tissue. Due to the low parasite infection rate typically observed in snails [42], the snails collected in 2017 were also subjected to PCR analyses to assess the presence of *F. hepatica* DNA and confirm the morphological identification of the snails following a protocol described in Graham-Brown et al., University of Liverpool (manuscript in preparation). Briefly, DNA was extracted from the entire snail using Chelex® method as described by Caron et al. [43]. The supernatant containing DNA was diluted 10 times with Tris-EDTA and stored at -20 °C until PCR.

A total of three PCR reactions were conducted for each snail. The first PCR targeted amplification of snail internal transcribed spacer 2 (snail ITS2) to confirm snail identify as *G. truncatula* [44], and also to act as an internal positive control, since snails are known to contain PCR inhibitors. Then the second and the third PCRs were used to determine *F. hepatica* infection status by targeting *F. hepatica* ITS2 (F hep ITS2), and *F. hepatica* cytochrome *c* oxidase subunit 1 (F hep *cox*1), respectively. F hep ITS2 was as described by Novobilsky et al. [45] and Caron et al. [46], and F hep *cox*1 was as described by Cucher et al. [47], with addition of 4 μg bovine serum albumin (BSA) in PCR mix and an increase of the PCR cycles to 40. The total volume of each PCR reaction was 25 μl, consisting of 4 μl 1:10 diluted template snail DNA, 12.5 μl Biomix^TM^ Red (Bioline, London, UK), 1μl (4 μg) BSA, 2 μl of 10 μM primer pairs in case of snail ITS2 PCR and F hep ITS2 PCR, and the rest made up with double distilled water. For F hep *cox*1 PCR, 0.5 μl of 5 μM primer pairs was used instead. Negative controls (double distilled water) and the following positive controls were included in each PCR reaction: 4 μl 1:10 diluted DNA extracted from *G. truncatula* infected with *F. hepatica,* 4 μl 1:10 diluted DNA extracted from non-infected *G .truncatula*, and 4 μl (0.1ng) of DNA extracted from adult *F. hepatica* tissue.

To confirm the snail identity based on shell morphology, representative samples of snail ITS2 PCR products were sequenced, i.e. four snails identified as *G. truncatula*, one snail identified as *Succinea putris* and one snail identified as *Radix balthica*. A snail was considered positive for *F. hepatica* infection if both F hep ITS2 and F hep *cox*1 PCRs amplified a product of the expected size (approximately 112 bp and 405 bp, respectively). All snails that had amplified a product for the F hep *cox*1 PCR were sequenced. The PCR products were purified using QIAquick PCR purification kit (Qiagen, Manchester, UK) and sequencing was performed by Source Bioscience (Nottingham, UK). Sequences were aligned using *Staden* package (preGAP4 version 1.6 and GAP4 version 11.2) and run through NCBI Nucleotide BLAST (http://blast.ncbi.nlm.nih.gov/) and compared with sequences available on GenBank.

## Results

### Analysis of longitudinal data

#### Summary of climate data

**Fig. 4:**
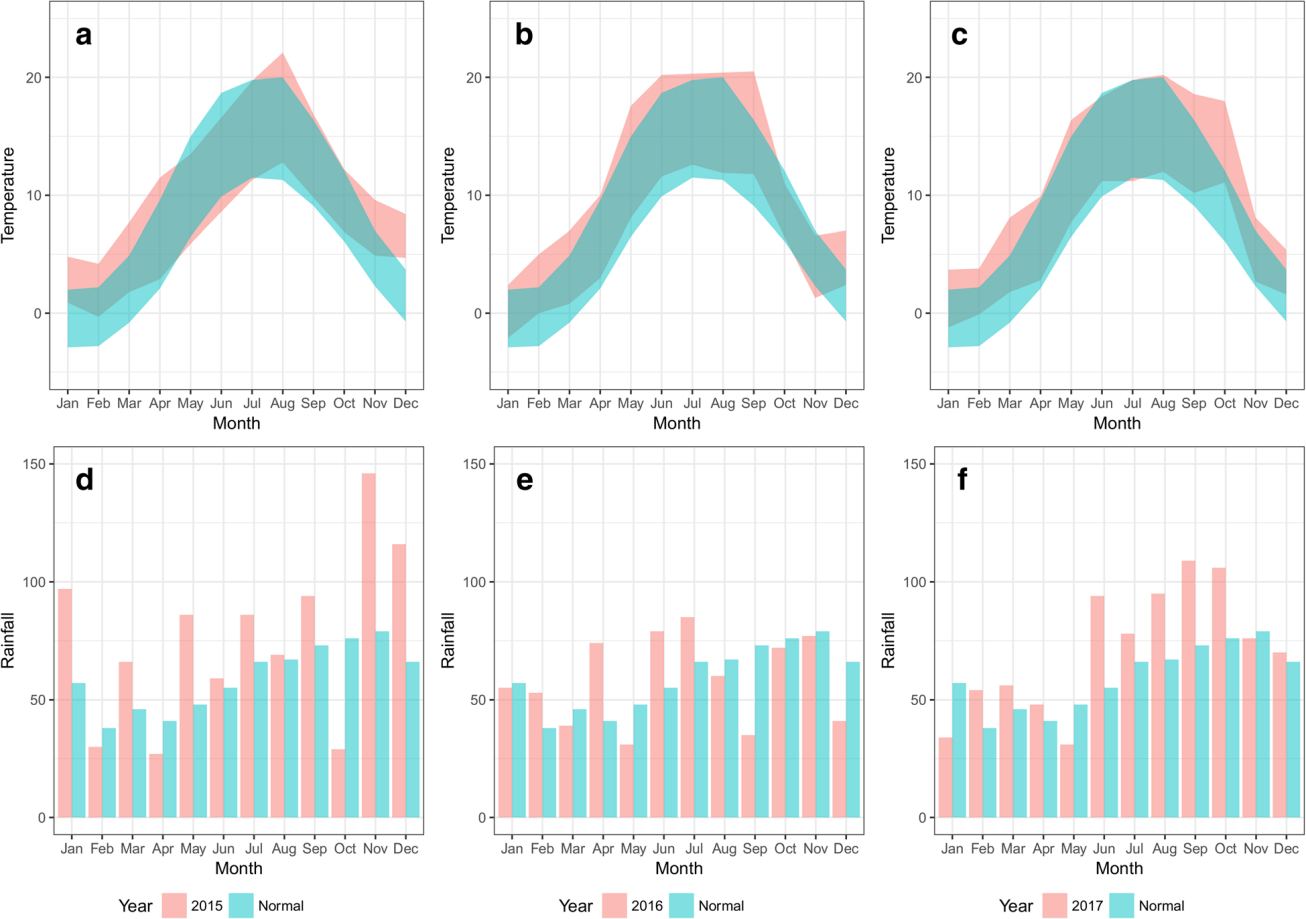
Danish climate data for the four farms for the study period (2015–2017: red) and 30 year average (1961–1990: blue). The climate in Denmark is a mixture of oceanic and continental temperate. The mean day highest and lowest temperatures of each month are shown above, while the total monthly precipitations are shown below

Climate data for the whole of Denmark for the study period as well as the 30-year average (1931–1960) are shown in Fig. 4. In general, the maximum air temperature exceeded 10 °C from April to October between 2015 and 2017 (Fig. 4a-c), although also in March 2015. The air temperature was highest in July and August 2015/2017, although air temperatures were high (*c*.20 °C) throughout June to September in 2016. The maximum and minimum temperatures during both winter periods (Nov 2015 - Mar 2016 and Dec 2016 - Mar 2017) were higher than the 30-year average. As for precipitation, 2016 was comparable to the 30-year average, while precipitation was very low in October 2015 followed by above average rainfall in November and December 2015 (Fig. 4d). Above average precipitation was also seen during June to October 2017 (Fig. 4f).

#### Graphs of overall individual animal data

**Fig. 5:**
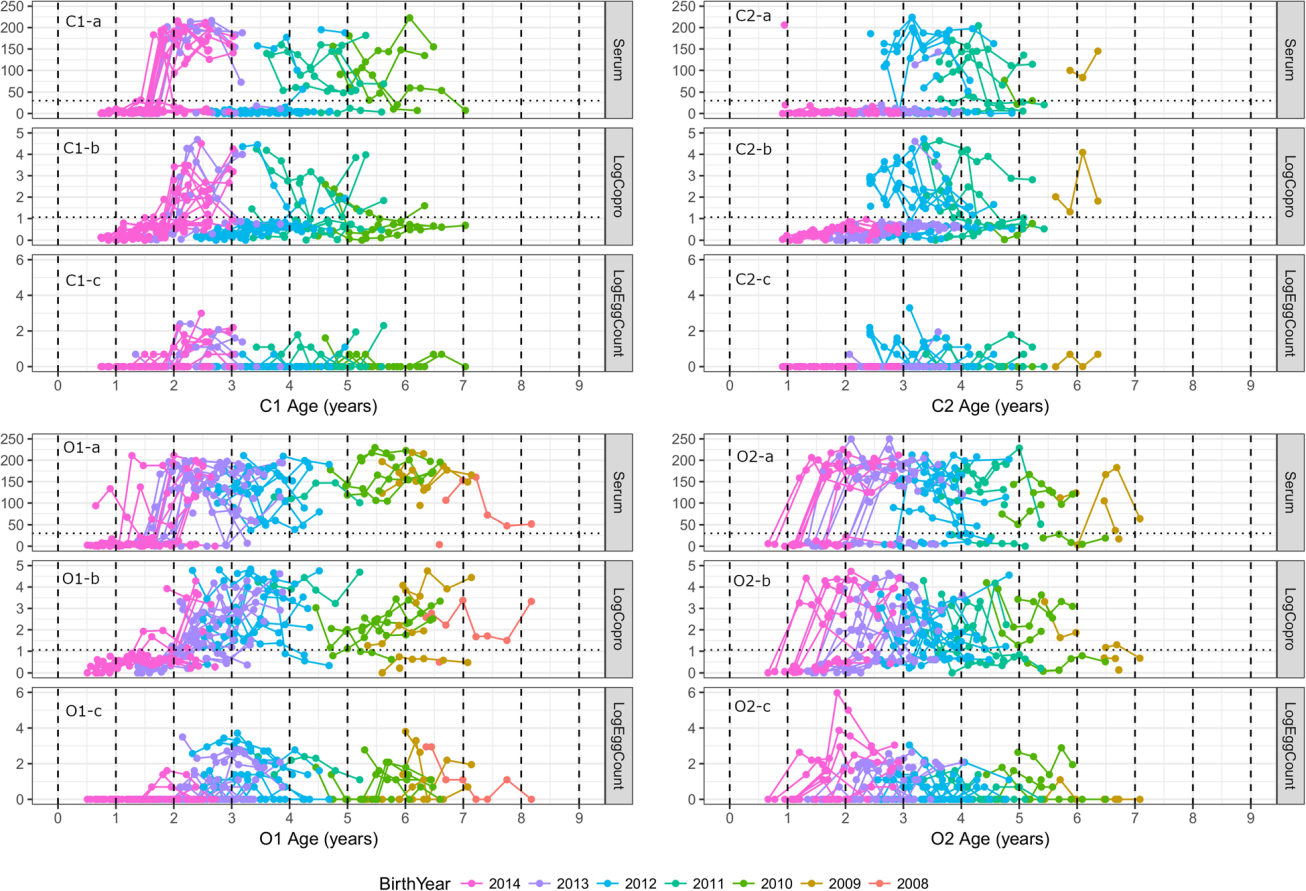
The summary of *F. hepatica* diagnostic test results according to farms and age during the study period (from spring 2015 to winter 2017). Colour indicates animals that were born in the same year. Coproantigen ELISA values are log-transformed (after adding a fixed constant of 1), and the cut-off defined as 1.89 (1.061 after transformation). Faecal egg counts in 5 g faeces were also log-transformed (after adding a fixed constant of 1) for the benefit of visualisation. Any post-treatment data are excluded

The raw data are plotted against the age of the animals according to diagnostic methods and farms in Fig. 5. On farm C1, most animals born during 2013 and 2014 seroconverted between the ages of 1.5 to 2 years (Fig. 5 C1-a). Animals over 4 years of age were also mostly seropositive, while a group of animals born in 2012 were seronegative throughout the study period. Copro-antigen values and FEC were positive from 2 years of age (Fig. 5 C1-b, c). On farm C2, no young animals seroconverted during the study period (Fig. 5 C2-a). Some animals born before 2012 had high serum and copro-antigen ELISA values, but only a small number of animals excreted liver fluke eggs (Fig. 5 C2-b, c). On farm O1, most young animals seroconverted by the age of 2 years (Fig. 5 O1-a). The older animals on this farm were all seropositive throughout the study period, and copro-antigen ELISA test from these animals were also positive, although the actual test values were variable (Fig. 5 O1-b). Copro-antigen values and FEC were positive from around 2 years of age, and high egg excretions were seen in both young and older animals (Fig. 5 O1-b, c). On farm O2, not all young animals seroconverted and the age at which young animals seroconverted was variable (Fig. 5 O2-a). High copro-antigen values and FEC were seen in animals younger than 2 years as well as in older animals (Fig. 5 O2-b, c).

Overall, the infection seemed to first occur when the animals were between 1–2 years of age on all four farms. The summary of raw data over the seven sampling days according to the farms and age cohorts is provided in Additional file 1: Figure S1. It should be noted that any post-treatment data have been excluded from Fig. 5 and Additional file 1.

#### Monthly BTM data

**Fig. 6:**
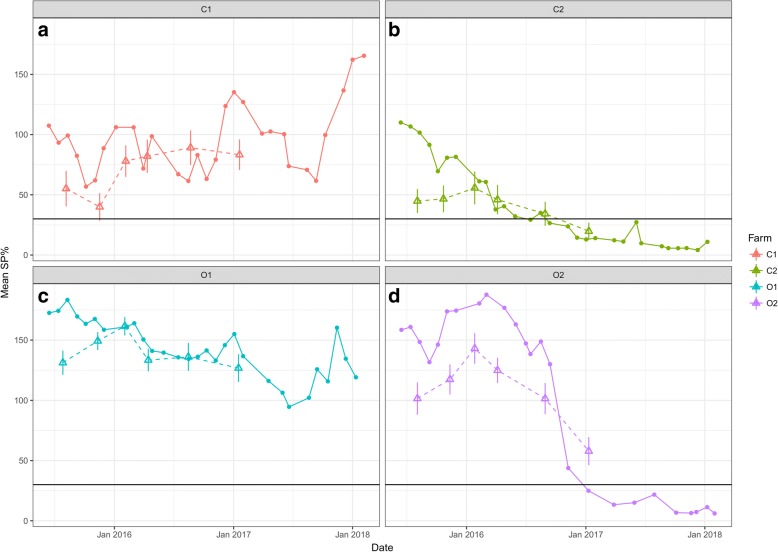
Monthly anti-*F. hepatica* antibody levels in bulk tank milk (BTM) (solid line) and average serum antibody levels of milking cows during the study period (triangle points with dashed line, error bars showing standard error of the mean) in the four farms

Antibody levels measured in BTM showed fluctuations during the study period (Fig. 6). On farms C2 and O2 a general decrease in BTM antibody levels was seen. The decrease was seen from the end of 2015 in C2 and from the end of 2016 in O2 (Fig. 6-b, d). BTM antibody levels corresponded well with the average serum antibody levels of milking cows. Pearson’s product moment correlation was *r*_(22)_ *=* 0.86 (95% CI: 0.70–0.94) and statistically significant (*P* < 0.0001).

#### GAMM analysis

**Fig. 7:**
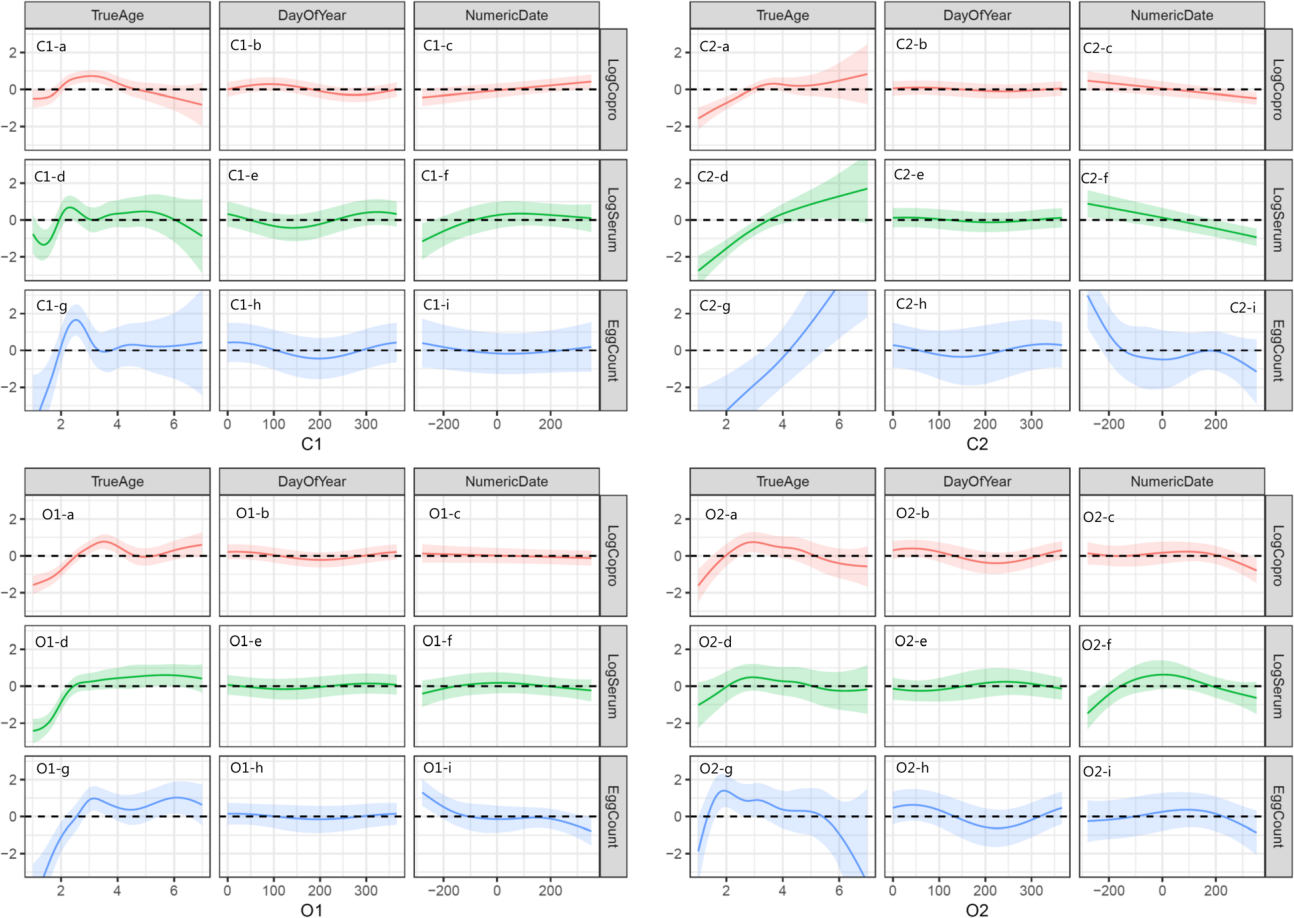
Results of generalised additive mixed models (GAMM) showing the relative effects of animal age, season of sampling, and date of the sampling (for longer-term temporal trends) within the studied farms. Each combination of farm and diagnostic test was modelled independently. The estimates are shown using solid lines and shaded areas indicate 95% confidence intervals. The y-axis is on the natural logarithm scale

The results of the separate GAMM models for each combination of farm and test type are shown in Fig. 7. Based on the model results, the animals’ age had a greater impact on the expected test results than either seasonality or the longer-term temporal effect associated with the farm as a whole on all four farms (Fig. 7a, d, g). In general, test values were low in very young animals, but peaked at the age of 2–4 years, and slowly declined as the animals got older, except for farm C2, where test values continued to increase with age. Differences in expected diagnostic test results through different seasons (time of year) were not substantial, but a small peak was observed later in the year for serum ELISA values (Fig. 7b), while peaks of copro-antigen ELISA and FEC occurred at the beginning of the year (Fig. 7e, h). Long-term temporal effects differed between the farms; farms C1 and O1 were relatively stable, while farms C2 and O2 (those using routine anthelmintics) showed a reduction at the end of the study period (Fig. 7c, f, i).

Approximate normality of residuals was observed in residual plots from all models. A small degree of residual temporal autocorrelation was observed in a small number of young animals from C1 and two animals from C2 on the copro-antigen models. Re-running the models without these observations gave qualitatively the same results, so the original models including all available data were retained.

#### Changes in test values post-treatment

Pre- and post-treatment data were available from 24 animals from O2 and 6 animals from C2. Of these, 17 animals (15 from O2 and 2 from C2) had high serum antibody levels pre-treatment and 12 of those converted to negative status within 195 days post-treatment (Additional file 2: Figure S2). Out of the remaining five animals, two animals were still highly positive (174.9 and 142.2 S/P%) at last sampling at 20 and 36 days post-treatment. Three others showed decreased, low antibody levels at last sampling (63.5, 43.8 and 65.7 S/P%), which were at 32, 132 and 148 days post-treatment, respectively. The copro-antigen ELISA results of 13 animals were positive just before treatment and all of these animals reverted to negative status at 20 to 85 days post-treatment, except for one animal. A sample collected 195 days post-treatment from this animal was nevertheless negative. The FEC of 12 animals were positive immediately before treatment and egg excretion was detected in only one of these animals 195 days post-treatment. In the other animals, *F. hepatica* eggs were not detected at 20 to 85 days post-treatment sampling.

### Comparison of diagnostic test results

The pairwise agreement between diagnostic methods at seven sampling times is summarised in Table 3. Good agreement was seen between serum ELISA and copro-antigen ELISA and moderate to good agreement was seen between copro-antigen ELISA and FEC. Only fair to moderate agreement was seen between serum ELISA and FEC. Agreement between serum ELISA and copro-antigen ELISA was highest in winter, while agreement between serum ELISA and FEC was highest during summer. The highest agreement between copro-antigen ELISA and FEC was seen during spring/summer period.

**Table 3:** Agreement between diagnostic tests (Cohen’s kappa) at each sampling time. Total number of observations is given in brackets, and any samples taken within 180 days of treatment are excluded

	Spring 2015	Summer 2015	Autumn 2015	Winter 2015/16	Spring 2016	Summer 2016	Winter 2016/17
Serum ELISA *vs* copro-antigen ELISA	0.794 (84)	0.747 (150)	0.571 (167)	0.830 (176)	0.807 (175)	0.721 (155)	0.811 (126)
Serum ELISA *vs* FEC	0.476 (87)	0.525 (150)	0.357 (167)	0.414 (175)	0.540 (175)	0.610 (155)	0.538 (126)
Copro-antigen ELISA *vs* FEC	0.710 (174)	0.641 (157)	0.520 (168)	0.539 (175)	0.662 (175)	0.771 (156)	0.574 (126)

There were 45 animals in the study that were slaughtered within 60 days after sampling and for which meat inspection data was therefore available. Of those, 10 animals were identified as liver fluke positive based on liver inspection at slaughter. The summary of the diagnostic tests results is shown in Additional file 3: Figure S3 and their pairwise Cohen’s kappa are summarised in Table 4.

**Table 4:** Cohen’s kappa statistics of pairwise comparisons of diagnostic tests for *F. hepatica* infection in animals (*n* = 45) that were slaughtered 7 to 60 days after the last sampling date during the study period

	Test positives	Cohen’s kappa pairwise comparison		
		Serum ELISA^a^	Copro-antigen ELISA	FEC
Liver inspection	22.2% (10/45)	0.287	0.245	0.267
Serum ELISA^a^	41.9% (18/43)	–	0.751	0.538
Copro-antigen ELISA	28.9% (13/45)	–	–	0.762
FEC	20% (9/45)	–	–	–

^a^2 missing values (*n* = 43)

### Snail surveys and detection of *Fasciola hepatica* in snails

In total, 301 *G. truncatula* snails were found on pastures used for grazing on the 4 farms. Other freshwater snails identified were *Lymnaea stagnalis* (Linnaeus, 1758), *Lymnaea palustris* (O.F. Müller, 1774), *Bythinia tentaculata* (Linnaeus, 1758), *Radix balthica* and number of specimens belonging to the genera *Planorbis*, *Succinea* and *Anisus* that could not be identified to species level based on shell morphology. In brief, the snails were found in typical habits such as riparian areas (along ditches), dense rush and water puddles created by heavy trampling, but a large number of snails were also observed within drinking troughs that were in use (Additional file 4: Table S1).

In June 2015, no *G. truncatula* was observed on two paddocks of farm C1: the home paddock for the milking cows and the pasture along a fjord for heifers (Fig. 2 C1-A/a, B and C). On farm C2, ten *G. truncatula* were found in June and another 13 specimens in October on the paddock used for heifers (Fig. 2 C2-A). On farm O1, three separate areas used for grazing were searched for snails (Fig. 3 O1-A, B, E, F). One *G. truncatula* was found on one of the two paddocks for young heifers (Fig. 3 O1-E) and another five were found on a marshland paddock grazed by larger heifers (Fig. 3 O1-F). On a similar, near-by marshland paddock used for grazing by farm O2, nine *G. truncatula* were found in October 2015, whereas no snails were observed on the home paddocks, and a more remote bog area also used for grazing (Fig. 3 O2-A, B, C, D). Shedding and dissection of the 38 collected *G. truncatula* in 2015 did not reveal any infection with *F. hepatica* or any other trematode parasites.

In October 2017, a total of 298 snails (263 *G. truncatula*, 33 *R. balthica*, one *S. putris*, and one terrestrial snail, which was not further identified) were retrieved from farms C1, O1, and O2 (farm C2 was not visited). A total of 246 *G. truncatula* were found in the fjord paddock on farm C1 (Fig. 2 C1-C; Additional file 4: Table S1). On farm O1, ten *G. truncatula* were obtained from the paddock for dry cows/in-heat heifers (Fig. 3 O1-B) and one additional *G. truncatula* from a home paddock for milking cows (a part of rotational grazing; Fig. 3 O1-A). On farm O2, six *G. truncatula* were detected in a ditch where milking cows were grazed (Fig. 3 O2-A), and 33 wandering snails (*R. balthica*, as identified by PCR) were collected from a water trough on another paddock grazed by milking cows (a part of rotational grazing; Fig. 3 O2-A). PCR products from the four snails morphologically identified as *G. truncatula* (344–411 bp) were 99–100% identical (E-values 0 or 2 × e^-178^) to *G. truncatula* sequences on GenBank (KT781267 and KF887031.1). Likewise, the identities of *S. putris* (168 bp) and *R. balthica* (356 bp) were verified by comparison with sequences from GenBank demonstrating 99% (MF148322.1, E-value: 1 × e^-76^) and 100% (LT623580.1, E-value: 0), respectively. The newly-generated sequences were deposited in the GenBank database under accession numbers MH561918-MH561923 (Additional file 4: Table S1).

Three out of 263 *G. truncatula* (1.1%) were found to be infected with *F. hepatica* by PCR. All the *F. hepatica* positive snails were *G. truncatula*: one from farm C1 found within dense rush, one from farm C1 found in a drinking trough, and one from farm O1 found within sparse rush (Additional file 4: Table S1). The sequences (348–353 bp) were 99–100% identical to *F. hepatica* sequence on GenBank (AF216697.1, E-values ranged from 0 to 2 × e^-177^). The sequences were deposited in GenBank under the accession numbers MH5619124-MH561926 (Additional file 4: Table S1).

## Discussion

Our study used intensive data collection from a number of different sources to investigate issues relevant to the control of *F. hepatica* on four Danish dairy farms. Each farm recruited for this study had critically different grazing management styles and the farmers had different attitudes towards *F. hepatica* control. However, despite these differences, there was a similar association between animal age and *F. hepatica* diagnosis across the four farms; infection tended to be acquired as young stock, although not necessarily in the first grazing season. This finding is consistent with our previous risk factor analysis, which showed heifers grazing on wet areas as a risk group and a predictor of farm status [25]. This was likely to be a reflection of the typical Danish practice, where younger calves and cows (with the exception of dry cows) are grazed close to the main farm buildings on relatively dry, high grounds, while heifers (and sometimes dry cows) are placed on marginal lands and allowed to graze freely for the entire grazing season [48, 49]. Indeed, many of the primiparous cows were already infected at the start of the study except for those from farm C1. This particular group of animals on farm C1 grazed on the same heifer paddock near the fjord (Fig. 2 C1-C) in 2014 without being infected. We speculate that flooding with seawater that occurred during winter 2013/2014 wiped out the snail population in that area, and animals consequently escaped liver fluke infection in this particular grazing season.

Most animals were infected before calving and carried the infection as they moved into the lactating herd. In older animals, interpretation of the diagnostic test results is a challenge, as it is unknown how long the parasite can live and how long the antibodies persist after elimination of the parasite. Ross [50] observed that most parasites were lost between 5th and 21st months after infection, while the remaining parasites could live at least 26 months. Based on our results, the longevity of the parasites could be longer than 26 months, as the multiparous cows from farm C2 that had no access to outdoor areas (except for dry cows in a sandy yard) or freshly-cut grass, were still seropositive at 4 years of age and over. Lasting antibodies after elimination of the parasites is a possible scenario, but antibody levels declined within 195 days post-treatment in the present study and similar findings have been seen in other studies [51, 52]. Additionally, copro-antigen ELISA values were above the cut-off and liver fluke eggs were present in the faeces in some of these older animals, indicating active infection.

If the parasites can persist for longer than two years, then positive results from cows in their third or higher lactation can either be a result of persistent infection or re-infection, which occurred most likely during the dry period. Dry cows are frequently grazed on marginal land together with heifers, and indeed our previous risk factor analysis showed odds of farm infection status was approximately four times higher if dry cows grazed on wet areas [25]. On farm C1, some multiparous animals over four years of age had low to moderate serum antibody levels and elevated copro-antigen ELISA levels, suggestive of potential reinfection on the pasture used for the lactating herd. However, the cohort of primiparous cows on farm C1 that were uninfected at the start of the study remained uninfected for the entire study period (Additional file 1: Figure S1). This suggests that the pasture used for the lactating cows constituted a minimal risk and that multiparous cows were probably carrying the parasites for years from the initial infection. In contrast, it seems that milking cows were continuously exposed to *F. hepatica* metacercariae on farm O1 because multiparous cows over four years of age showed continuously high serum ELISA values (> 100 S/P%) and many of their copro-antigen ELISA values were well above the cut-off. In addition, while most multiparous cows on farm C1 did not excrete any *F. hepatica* eggs in the faeces, egg excretion was observed in a number of multiparous cows on farm O1. This coincides with the findings of Ross [50] that egg laying capacity of the fluke was maximal at 3–8 months post-infection, but reduced to low or negative faecal egg counts during the chronic phase of infection (> 10 months post-infection). Mezo et al. [53] also documented that animals infected with low fluke burden showed low antibody responses during the chronic phase of *F. hepatica* infection. Although Knubben-Schweizer et al. [5] recommended serological testing of the oldest animals in the herd to determine infection in the milking cows, our conclusion is that the assessment of whether the lactating herd is re-infected by *F. hepatica* or not, is difficult solely based on serum ELISA dichotomised into positive or negative results. The serum ELISA could be high with continuous exposure, while long-lasting infection may have low to moderate serum ELISA levels. However, to document/confirm re-infection within the milking herd, it should be complemented with either copro-antigen ELISA or faecal egg counts.

The temporal patterns of infection differed greatly among the four farms over the study period. This is likely to be due in part to different grazing management, but also relate to the introduction of regular treatments against *F. hepatica* on two of the farms in 2016, which was, of course, influenced by our consultations with the farmers on the findings during the study period. BTM ELISA showed a good correlation with average serum antibody levels of milking cows, and the overall progression of the disease was clearly seen from the BTM ELISA results (Fig. 6). The two organic farms had high infection levels shown by BTM ELISA compared to the two conventional farms at the start of the study. During the study, two farms initiated *F. hepatica* control by treatment (heifers at housing on farm C2 and dry cows and heifers pre-calving on farm O2) and grazing management, resulting in decreased level of *F. hepatica* infection at the end of the study. This was also reflected in the decreasing longer-term temporal trend estimated by the GAMM on farms C2 and O2 (Fig. 7). Farm C1 also started treatment of heifers pre-calving in 2017, but BTM ELISA showed an increased level of infection from late 2017 (Fig. 6). This was unexpected, based on the assumption that re-infection was unlikely to occur on the permanent paddock for the lactating cows. However, the second half of 2017 was wetter than normal (20–30% more rain) (Fig. 1), and therefore transmission of *F. hepatica* on the lactating cow paddock (Fig. 2 C1-A/a) may have occurred in 2017.

According to the GAMM, seasonality did not seem to be as strongly associated with the test values compared to age and the longer-term temporal trends within the farms. Considering the relatively long-lasting nature of infection, it is not unexpected that test values are relatively stable between seasons after accounting for the effects of age and longer-term temporal trends. However, modest fluctuations according to seasons were seen, and generally speaking, the peak occurred first for serum ELISA in autumn, followed by copro-antigen ELISA and FEC (Fig. 5). Agreement between serum ELISA and copro-antigen ELISA was also highest in winter, while agreement between serum ELISA and FEC was highest in summer. This reflects the fact that the three diagnostic tests differ in the time of detection; serum ELISA can detect infection within 2–4 weeks post-infection [54], while copro-antigen ELISA values rises 6–8 weeks post-infection in cattle [55]. This has an important connotation for the timing of sampling in order to diagnose *F. hepatica* on a farm. Serum ELISA can be used to test for *F. hepatica* exposure at housing in the autumn, whereas under-diagnosis is likely to occur if copro-antigen ELISA or FEC is used at that time of the year.

It has been speculated that as a result of climate change, release of metacercariae in spring from overwintered snails, may become more significant as a source of infection for grazing animals [5]. There was little indication of this in our study. Some animals sero-converted by summer in 2015, but as our sampling time was end July to August, the infection could have been acquired either early in the grazing season (winter infection) or just before the summer sampling (summer infection). However, no increase in copro-antigen ELISA values or egg excretion was observed from these animals, suggesting that infection occurred mid-summer and therefore that summer infection is still the most relevant to consider for cattle in Denmark.

Three out of 263 *G. truncatula* (1.1%) were found to be infected with *F. hepatica* by PCR. Prevalences of *F. hepatica* in *G. truncatula* reported from previous studies differ substantially from 0.5% to 82% [21, 56, 57], although large studies conducted in France found around 5–12.5% of the snails infected [58, 59]. Significant differences in *F. hepatica* prevalence in snails have been found to be associated with differences in seasons, locations, and year of the study [18, 57, 59]. Likewise, we found great inter-annual variation in the snail survey results; many snails were found in October 2017 compared to June-October 2015. As evident from Fig. 4, many temporary water bodies especially on the fields around the farms were dried out due to scarce rainfall in October 2015, while potential habitats were expanded in October 2017 due to high rainfall. This highlights the effect of climatic factors on snail habitats and also the importance of frequent samplings/observations to avoid false negative findings. Nevertheless, we confirmed over time the presence of snails on many of the pastures where we suspected transmission took place. It is perhaps noteworthy that a positive snail was recovered from a drinking trough. It is known that floating metacercariae can form on the surface of the water after cercariae exit the snail [60], and thus transmission through infected drinking water is possible. These authors considered the transmission route by the floating metacercariae to be unimportant, as dispersal and survival of floating metacercariae was low in running water under both laboratory and field conditions. Yet the authors mentioned that metacercariae could float for over three months on the surface of stagnant water, and therefore the presence of an infected snail in a water trough without a pump or a tap could become a source of *F. hepatica* infection. Overall, this study demonstrated some of the difficulties related to detection of snails and snail habitats. Unless a clear, quick and easy guideline is developed, precise identification of snail habitats (as transmission sites) is unlikely to be accepted as part of practical control programs. Recent developments in environmental DNA (eDNA) based methods to detect *G. truncatula* and *F. hepatica* directly in the environment [61], as well as the use of drone imagery to delineate potential snail habitats [62] could provide a future avenue, given that these methods become sufficiently easy and cheap.

Based on our results we suggest improved practical guidelines for diagnosis and management of fasciolosis on dairy farms with grazing stock. First, it is important to determine whether transmission is taking place in the young stock only (e.g. farm C2) or both in young stock and older cows (e.g. farm O1). This pattern of infection is again related to whether they graze contaminated pastures or not. We therefore recommend that identification of contaminated pasture is assessed by taking representative serological samples from planned second-year grazers and from cows older than third lactation (or the oldest cows) before turn-out. In addition, faecal samples from cows should preferably be analysed by copro-antigen ELISA or FEC to confirm active infection. Positive samples suggest that the pasture used to graze this cohort of animals during the previous summer was contaminated with metacercariae. The procedure should be repeated at housing for young stock, if they were negative at turn-out, to determine if they have picked up infection over the summer grazing period. If animals are grazed on different pastures, representative samples from each group should be taken. Once age groups at risk is clarified and fluke risk paddocks are identified, medicinal and non-medicinal control can be tailored and applied depending on the farmer’s motivation and capabilities as suggested by Knubben-Schweizer et al. [5]. However, as demonstrated in farms O1 and O2, some farms have very limited options for non-medicinal control as the majority of pastures have extensive wet areas suitable for the intermediate host snails. Efficacious treatment with triclabendazole during the dry period was a challenge to farmers due to restrictions related to expected calving, particularly on organic farms with long withdrawal periods. After implementation of the control program, progress can be monitored by BTM ELISA, preferably in spring when the antibody levels are highest. The detailed diagnosis of individual animals may need to be repeated in order to reduce the impact of year-to-year variation within the same farm.

## Conclusions

This longitudinal study on four dairy farms in Denmark showed that the patterns of *F. hepatica* infection varied considerably between farms due to different grazing management (e.g. snail habitats) and anthelmintic strategies employed. Careful interpretation was required based on the grazing history of the animals in the context of precipitation (climate), as year-to-year variation was also evident. However, some commonalities were seen despite these differences; in particular heifers were the main risk group for *F. hepatica* infection on all the farms. On two farms old cows had persistent infections derived from initial infection as heifers, while lactating cows were continuously exposed (most likely as dry cows) to metacercariae on one of the other farms. We conclude that the adoption of a stringent treatment schedule of pre-calving heifers when there is no transmission in the lactating cow herd (housed or on non-risk pasture) can lead to lower BTM ELISA values, indicative of reduced exposure to *F. hepatica*. If there is transmission in the lactating cow herd, consistent dry cow treatments can reduce the prevalence. However, such an intensive treatment program may not readily be accepted by organic producers, and further studies are required to demonstrate if non-medicinal approach (e.g. genetically robust breeding lines, a more precise spatiotemporal delineation of pasture risk areas and/or biological control of snails) in a longer perspective can limit the requirement of anthelmintic treatments.

## Additional files

Additional file 1: **Figure S1.** Summary of raw data according to farms, cohort groups, and *F. hepatica* diagnostic test results. (Cohort 1 is the youngest group, and cohort 4 is the oldest animals). Serum ELISA values are not log-transformed and the cut-off is defined as 30. Coproantigen ELISA values are log-transformed (after adding a fixed constant of 1) and cut-off defined as 1.89 (1.061 after transformation). Faecal egg counts in 5 g faeces were also log-transformed (after adding a fixed constant of 1) for the benefit of visualisation. Any post-treatment data were excluded. The samples from same animals are connected with solid lines and the pink shows the average value of each sampling point within the cohorts. (TIFF 2812 kb)

Additional file 2: **Figure S2.** The antibody response after treatment in 17 animals. Day 0 is the day of treatment. (TIFF 723 kb)

Additional file 3: **Figure S3.** The results of diagnostic tests for *F. hepatica* infection in animals (*n* = 43, as two serum samples were missing) that were slaughtered 7 to 60 days after the last sampling date during the study period (1, liver condemnation; 0, no liver condemnation). (TIFF 2812 kb)

Additional file 4: **Table S1.** Summary of snails collected in 2017 that were analysed by PCR for snail species and *F. hepatica* infection status. (XLSX 11 kb)

## Abbreviations

bp: base pair
BTM: Bulk tank milk
*cox*1: cytochrome *c* oxidase subunit 1
DCD: Danish cattle database
DH: Danish Holsten
DNA: Deoxyribonucleic acid
ELISA: Enzyme-linked immunosorbant assay
EU: ELISA unit
FEC: Faecal egg count
GAMM: generalised additive mixed model
ITS-2: internal transcribed spacer 2
PCR: polymerase chain reaction
S/P%: Sample to positive percentage
