# Supplementary figures and images for: Patterns of Fasciola hepatica infection in Danish dairy cattle: implications for on-farm control of the parasite based on different diagnostic methods
Source: Parasit Vectors. 2018 Dec 29;11:674. doi: 10.1186/s13071-018-3248-z (PMC6310998; doi:10.1186/s13071-018-3248-z)

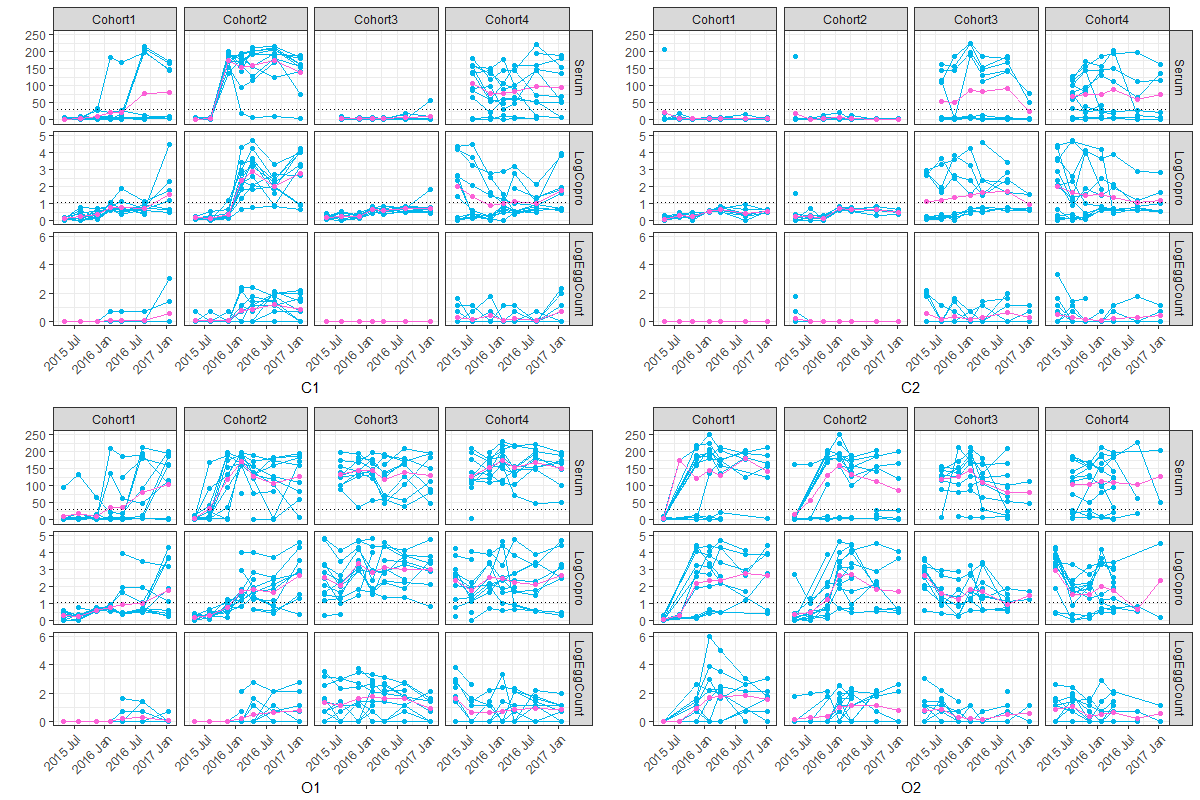

Supplement: Supplementary file 1 — Figure S1. Summary of raw data according to farms, cohort groups, and F. hepatica diagnostic test results. (Cohort 1 is the youngest group, and cohort 4 is the oldest animals). Serum ELISA values are not log-transformed and the cut-off is defined as 30. Coproantigen ELISA values are log-transformed (after adding a fixed constant of 1) and cut-off defined as 1.89 (1.061 after transformation). Faecal egg counts in 5 g faeces were also log-transformed (after adding a fixed constant of 1) for the benefit of visualisation. Any post-treatment data were excluded. The samples from same animals are connected with solid lines and the pink shows the average value of each sampling point within the cohorts. (TIFF 2812 kb) [file 13071_2018_3248_MOESM1_ESM.tiff]

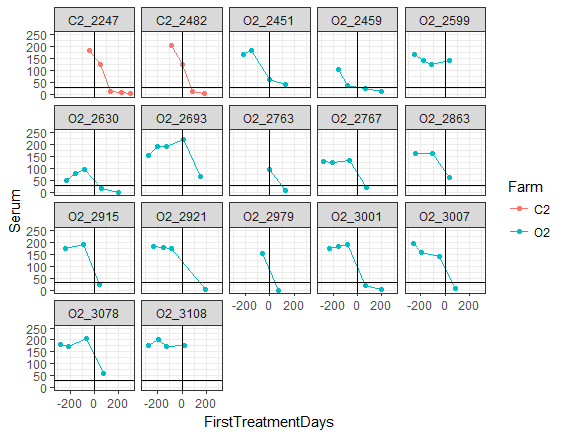

Supplement: Supplementary file 2 — Figure S2. The antibody response after treatment in 17 animals. Day 0 is the day of treatment. (TIFF 723 kb) [file 13071_2018_3248_MOESM2_ESM.tiff]

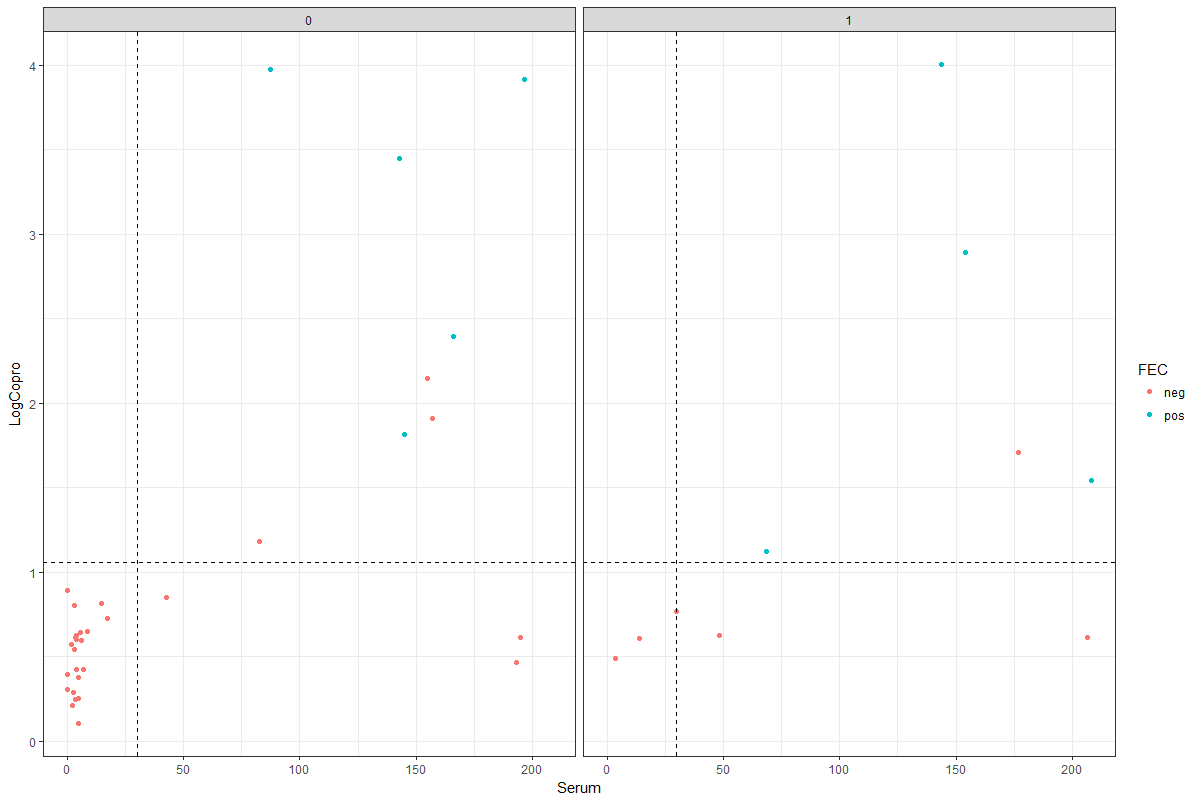

Supplement: Supplementary file 3 — Figure S3. The results of diagnostic tests for F. hepatica infection in animals (n = 43, as two serum samples were missing) that were slaughtered 7 to 60 days after the last sampling date during the study period (1, liver condemnation; 0, no liver condemnation). (TIFF 2812 kb) [file 13071_2018_3248_MOESM3_ESM.tiff]
